# Carrier frequency estimation of pathogenic variants of autosomal recessive and X-linked recessive mendelian disorders using exome sequencing data in 1,642 Thais

**DOI:** 10.1186/s12920-023-01771-w

**Published:** 2024-01-02

**Authors:** Wanna Chetruengchai, Prasit Phowthongkum, Vorasuk Shotelersuk

**Affiliations:** 1 Excellence Center for Genomics and Precision Medicine, King Chulalongkorn Memorial Hospital, the Thai Red Cross Society, Bangkok, 10330 Thailand; 2 Center of Excellence for Medical Genomics, Medical Genomics Cluster, Department of Pediatrics, Faculty of Medicine, Chulalongkorn University, Bangkok, 10330 Thailand; 3 Division of Medical Genetics and Genomics, Department of Medicine, Faculty of Medicine, Chulalongkorn University, Bangkok, Thailand

**Keywords:** Carrier screening, WES, Thais

## Abstract

**Background:**

People with autosomal recessive disorders often were born without awareness of the carrier status of their parents. The American College of Medical Genetics and Genomics (ACMG) recommends screening 113 genes known to cause autosomal recessive and X-linked conditions in couples seeking to learn about their risk of having children with these disorders to have an appropriate reproductive plan.

**Methods:**

We analyzed the exome sequencing data of 1,642 unrelated Thai individuals to identify the pathogenic variant (PV) frequencies in genes recommended by ACMG.

**Results:**

In the 113 ACMG-recommended genes, 165 PV and likely PVs in 60 genes of 559 exomes (34%, 559/1642) were identified. The carrier rate was increased to 39% when glucose-6-phosphate dehydrogenase (*G6PD*) was added. The carrier rate was still as high as 14.7% when thalassemia and hemoglobinopathies were excluded. In addition to thalassemia, hemoglobinopathies, and G6PD deficiency, carrier frequencies of > 1% were found for Gaucher disease, primary hyperoxaluria, Pendred syndrome, and Wilson disease. Nearly 2% of the couples were at risk of having offsprings with the tested autosomal recessive conditions.

**Conclusions:**

Based on the study samples, the expanded carrier screening, which specifically targeted common autosomal recessive conditions in Thai individuals, will benefit clinical outcomes, regarding preconception/prenatal genetic carrier screening.

**Supplementary Information:**

The online version contains supplementary material available at 10.1186/s12920-023-01771-w.

## Background

Although each Mendelian disease is generally uncommon, with > 5,000 known heritable disorders, they are accounted for ~ 20% of infant mortality, ~ 18% of pediatric hospitalizations, and substantial numbers in adults in the United States [1]. At least 2,000 autosomal recessive diseases [2] and 500 X-linked diseases [3] have been identified. The preconception carrier screening for these disorders and proper reproductive option counseling have been shown to be cost-effective measures that reduce the burden of Mendelian diseases [4–7]. The studies of the carrier rate of these disorders are highly varied with the number of tested genes and the genes included in the studies among different populations with no consensus agreement [8–11]. Until recently, the American College of Medical Genetics and Genomics (ACMG) released a set of 113 genes, both autosomal recessive and X-linked conditions, proposed as a standard list of genes for carrier screening [12]. Because X-linked glucose-6-phosphate dehydrogenase (G6PD) deficiency is the most common genetic disorder in Thailand and Southeast Asia [13], we added *G6PD* into the list and determined the carrier frequency and variant distribution of 114 recessive genes in the Thai population using the exomes of 1,642 unrelated Thais.

## Methods

### Study samples

In our study, we collected a sample of 1642 exomes from unrelated healthy Thai individuals, consisting of 811 men and 831 women. Prior to participation, informed consent was obtained from each individual. The study was conducted in accordance with ethical guidelines and was approved by the Institutional Review Board of the Faculty of Medicine, Chulalongkorn University (IRB No. 264/62). Our research with human subjects conforms with the Declaration of Helsinki (1964). The data analyzed in our study were part of the Thai Reference Exome database, although not all data from the database were used for our analysis [14]. It is important to note that for the patient cohort, we specifically excluded variants in genes known to be responsible for the patients' diagnosed conditions. This ensured that our analysis focused on carriers of pathogenic variants unrelated to the diagnosed diseases in the patient cohort.

### Sequencing and bioinformatics analysis

Genomic DNA was extracted from peripheral blood leukocytes. Exomes were captured using either a TruSeq® Exome Kit (Illumina, San Diego, CA) on the NextSeq 500 system or SureSelect Human All Exons (Agilent Inc., Santa Clara, CA) on the Hiseq 4000 and Novaseq 6000 Systems. The reagents are according to the manufacturer's standard protocol. Sequences were aligned to the human reference genome (GRCh37) using the Burrows–Wheeler Aligner package version 0.7.15 [15]. Variant calling was performed using the Genome Analysis Tool Kit (GATK Best Practice V3.7; Broad Institute), which is called by HaplotypeCaller [16]. ANNOVAR was used to annotate genetic variants. The variant interpretation was limited to the 113 ACMG-recommended genes and *G6PD*. We included variants listed as PV and LPV according to VarSome's ACMG classification [17] with a final manual curation by the authors.

### Statistical analysis

The frequency and 95% confidence interval were calculated using the proportion of carriers in total individuals tested (n/1642) and the Wilson method, respectively.

## Results

Using 1,642 exome sequencing data of unaffected parents whose children had rare diseases, we first excluded pathogenic and likely pathogenic variants (PV and LPV, respectively) found in these exomes, which caused the diseases in their children. We excluded 13 PV and LPV in 18 exomes (1%, 18/1642) (S1 Table).

**Fig. 1:**
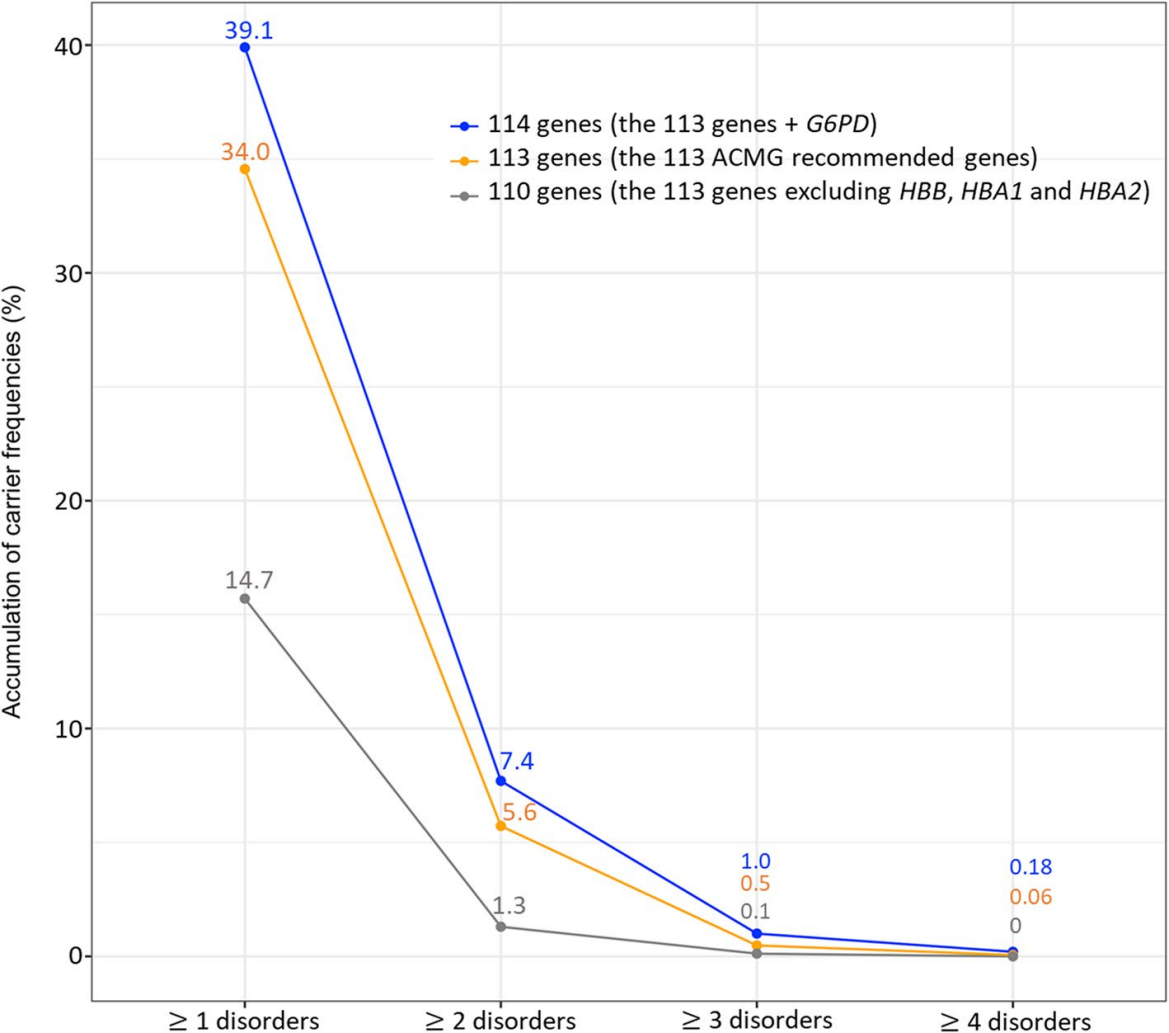
Carrier frequencies of one or more genetic disorders in 114 genes of 1,642 unrelated Thais. Orange color represents carrier frequencies in 113 ACMG recommended genes. Blue color represents those in 114 genes, which are the 113 genes and G6PD. Gray color represents those in 110 genes, which are the 113 genes excluding three genes underlying thalassemia and hemoglobinopathies (*HBB*, *HBA1*, and *HBA2*)

Of the 113 ACMG-recommended genes, 165 PV and LPV in 60 genes of 559 exomes were found (559/1642: 34%). For *G6PD*, 7 PV and LPV of 127 exomes (female) were identified. In the analysis of a total of 114 genes, 172 PV and LPV in 61 genes of 640 exomes (640/1642: 39%) were identified (Table 1). Although we excluded *HBA1*, *HBA2*, and *HBB* genes causing thalassemia and hemoglobinopathies, the carrier rate of at least one screened disorder was still as high as 14.7% (241/1642) in our cohort. Of the samples, 7.4% (121/1642) carried more than one PV and LPV (Fig. 1 and S2 Table).

**Table 1:** Carrier frequencies of genetic diseases identified in 114 genes of 1,642 unrelated Thai individuals (Bold text: carrier frequency ≥ 1/250)

Disease group (Number of individuals)	Gene	Phenotype disease	Mode of inheritance	Number of Individual	1 in	Carrier frequency (%) (Wilson 95% Interval)
**1.Hemolytic disorders with positive selection from malaria (518)**	***HBB***	**β-thalassemia**	**AR**	**321**	**6**	**19.55 (17.7–21.54)**
	***G6PD***	**Glucose-6-phosphate dehydrogenase deficiency**	**X-linked**	**127**	**13**^**a**^	**7.73 (6.5–9.1)**^**a**^
	***HBA2***	**ɑ-Thalassemia1**	**AR**	**65**	**26**	**3.96 (3.12–5.01)**
	*HBA1*	ɑ-Thalassemia2	AR	5	329	0.3 (0.13–0.7)
**2.Inherited metabolic disorders (142)**	***GBA***	**Gaucher disease, type I**	**AR**	**27**	**61**	**1.64 (1.13–2.38)**
	***AGXT***	**Hyperoxaluria, primary type I, II**	**AR**	**18**	**92**	**1.1 (0.7–1.73)**
	***ATP7B***	**Wilson disease**	**AR**	**17**	**97**	**1.04 (0.65–1.66)**
	***PAH***	**Phenylketonuria**	**AR**	**13**	**127**	**0.79 (0.46–1.35)**
	***CYP27A1***	**Cerebrotendinous xanthomatosis**	**AR**	**7**	**235**	**0.43 (0.21–0.88)**
	*ABCD1*	Adrenoleukodystrophy (ALD)	X-linked	5	329^a^	0.3 (0.13–0.71)^a^
	*GAA*	Glycogen storage disease, type II (Pompe disease)	AR	4	411	0.24 (0.09–0.62)
	*PMM2*	Carbohydrate-deficient glycoprotein syndrome type Ia	AR	4	411	0.24 (0.09–0.62)
	*TYR*	Oculocutaneous albinism type 1A and 1B	AR	4	411	0.24 (0.09–0.62)
	*ALPL*	Hypophosphatasia, adult, childhood and infantile	AR	3	548	0.18 (0.06–0.53)
	*FMO3*	Trimethylaminuria	AR	3	548	0.18 (0.06–0.53)
	*MMACHC*	Methylmalonic aciduria with homocystinuria cblC type	AR	3	548	0.18 (0.06–0.53)
	*ARSA*	Metachromatic leukodystrophy	AR	2	821	0.12 (0.03–0.44)
	*CBS*	Homocystinuria, B6 responsive and nonresponsive	AR	2	821	0.12 (0.03–0.44)
	*DHCR7*	Smith–Lemli–Opitz syndrome	AR	2	821	0.12 (0.03–0.44)
	*DLD*	Dihydrolipoamide dehydrogenase deficiency	AR	2	821	0.12 (0.03–0.44)
	*FAH*	Tyrosinemia type I	AR	2	821	0.12 (0.03–0.44)
	*FKRP*	Muscular dystrophy–dystroglycanopathy, type A, 5, type B, 5	AR	2	821	0.12 (0.03–0.44)
	*FKTN*	Cardiomyopathy;dilated;1X, Walker–Warburg congenital muscular dystrophy	AR	2	821	0.12 (0.03–0.44)
	*GALT*	Galactosemia	AR	2	821	0.12 (0.03–0.44)
	*GBE1*	Glycogen storage disease;type IV, GBE1-related disorders	AR	2	821	0.12 (0.03–0.44)
	*MMUT*	Methylmalonic aciduria–methylmalonyl–CoA mutase deficiency	AR	2	821	0.12 (0.03–0.44)
	*POLG*	Mitochondrial DNA depletion syndrome 4A,4B	AR	2	821	0.12 (0.03–0.44)
	*ACADM*	Medium-chain acyl-coenzyme A dehydrogenase deficiency	AR	1	1642	0.06 (0.01–0.34)
	*ACAT1*	ɑ-Methylacetoacetic aciduria	AR	1	1642	0.06 (0.01–0.34)
	*ALDOB*	Hereditary fructosuria	AR	1	1642	0.06 (0.01–0.34)
	*BTD*	Biotinidase deficiency	AR	1	1642	0.06 (0.01–0.34)
	*CPT2*	Carnitine palmitoyltransferase II deficiency, infantile, lethal neonatal	AR	1	1642	0.06 (0.01–0.34)
	*GNPTAB*	Mucolipidosis type II alpha/beta	AR	1	1642	0.06 (0.01–0.34)
	*HEXA*	Tay–Sachs disease	AR	1	1642	0.06 (0.01–0.34)
	*HPS1*	Hermansky Pudlak S. 1	AR	1	1642	0.06 (0.01–0.34)
	*IDUA*	Mucopolysaccharidosis, Ih (Hurler S), Ih/s (Hurler–Scheie S)	AR	1	1642	0.06 (0.01–0.34)
	*MCCC2*	3-methylcrotonyl CoA carboxylase 2 deficiency	AR	1	1642	0.06 (0.01–0.34)
	*OTC*	Ornithine transcarbamylase deficiency	X-linked	1	1642^a^	0.06 (0.01–0.34)^a^
	*RARS2*	Pontocerebellar hypoplasia type 6	AR	1	1642	0.06 (0.01–0.34)
**3.Other (123)**	***SLC26A4***	**Deafness autosomal recessive 4, Pendred syndrome**	**AR**	**18**	**92**	**1.1 (0.7–1.73)**
	***CFTR***	**Cystic fibrosis**	**AR**	**16**	**103**	**0.97 (0.6–1.58)**
	***USH2A***	**Usher syndrome, type 2A**	**AR**	**16**	**103**	**0.97 (0.6–1.57)**
	***NEB***	**Nemaline myopathy 2**	**AR**	**12**	**137**	**0.73 (0.42–1.27)**
	***GJB2***	**Nonsyndromic hearing loss recessive 1A, Nonsyndromic hearing loss dominant 3A**	**AR**	**9**	**183**	**0.55 (0.29–1.04)**
	***MCPH1***	**Primary microcephaly 1, recessive**	**AR**	**8**	**206**	**0.49 (0.25–0.96)**
	***CEP290***	**Joubert syndrome 5, Leber congenital amaurosis 10**	**AR**	**7**	**235**	**0.43 (0.21–0.88)**
	***CLCN1***	**Congenital myotonia, autosomal recessive form**	**AR**	**7**	**235**	**0.43 (0.21–0.88)**
	***PKHD1***	**Autosomal recessive polycystic kidney disease**	**AR**	**7**	**235**	**0.43 (0.21–0.88)**
	*AIRE*	Autoimmune polyendocrinopathy syndrome type I	AR	5	329	0.3 (0.13–0.7)
	*BBS2*	Bardet–Biedl syndrome 2, Retinitis pigmentosa 74	AR	3	548	0.18 (0.06–0.53)
	*EVC2*	Chondroectodermal dysplasia	AR	3	548	0.18 (0.06–0.53)
	*ABCC8*	Diabetes mellitus, permanent neonatal 3	AR	2	821	0.12 (0.03–0.44)
	*CC2D2A*	Joubert syndrome 9, Meckel syndrome 6	AR	2	821	0.12 (0.03–0.44)
	*DYNC2H1*	thoracic dysplasia 3 with or without polydactyly	AR	2	821	0.12 (0.03–0.44)
	*ERCC2*	Cerebrooculofacioskeletal syndrome 2, Trichothiodystrophy 1;photosensitive	AR	1	1642	0.06 (0.01–0.34)
	*FANCC*	Fanconi anemia, complementation group C	AR	1	1642	0.06 (0.01–0.34)
	*NPHS1*	Finnish congenital nephrotic syndrome	AR	1	1642	0.06 (0.01–0.34)
	*PCDH15*	Deafness, autosomal recessive 23,Usher syndrome;type 1F	AR	1	1642	0.06 (0.01–0.34)
	*PRF1*	Hemophagocytic lymphohistiocytosis, familial, 2	AR	1	1642	0.06 (0.01–0.34)
	*XPC*	Xeroderma pigmentosum	AR	1	1642	0.06 (0.01–0.34)

^a^For X-linked disorders, the carrier frequencies are calculated based on the total number of female individuals which is 831 out of 1,642 individuals in this study

### Gene carrier rate

In our cohort, all top three gene carrier rates were in hemolytic disorders with positive selection from malaria. The gene with the highest carrier rate is *HBB*, with 50.2% (321/640) and 19.6% (291/1642) of all carriers and participants, respectively. The second most common carrier gene is *G6PD*, with 19.8% (127/640) and 7.7% (127/1642) of all carriers and participants, respectively. *HBA2* is the third most common gene, with 10.2% (65/640) and 4% (65/1642) of all carriers and participants, respectively.

**Fig. 2:**
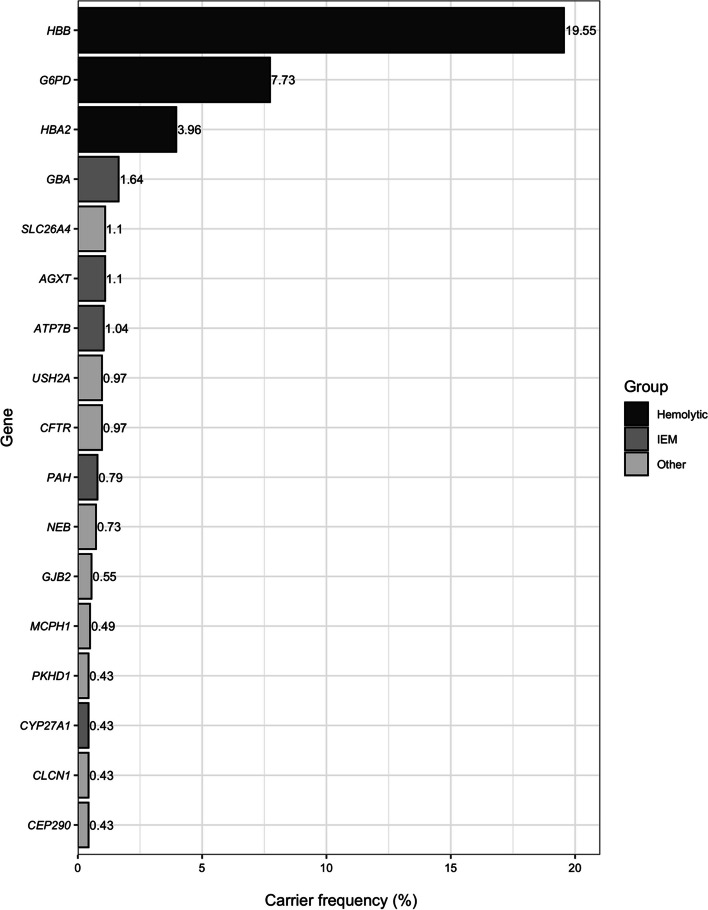
The 17 genes underlying autosomal recessive disorders with carrier frequencies of ≥ 1/250 (0.4%) in the 1,642 unrelated Thai individuals

There were 17 autosomal recessive genes with carrier frequencies of at least 1/250, an expected prevalence at birth of at least 1 in 250,000. As shown in Fig. 2, three, five, and nine of these genes are related to hemolytic disorders (*G6PD*, *HBB*, and *HBA2*), inborn errors of metabolism (*AGXT*, *ATP7B*, *CYP27A1*, *GBA*, and *PAH*), and in the miscellaneous group (*CEP290*, *CFTR*, *CLCN1*, *GJB2*, *MCPH1*, *NEB*, *PKHD1*, *SLC26A4*, and *USH2A*), respectively.

### Variant carrier rate

The most common PV is *HBB*; c.79G > A (p.Glu27Lys) responsible for hemoglobin E (HbE), representing 45.5% (291/640) and 17.7% (291/1642) of all carriers and participants, respectively. *HBA2*; c.427 T > C (p. Ter143Glnext*31, also known as Hb Constant Spring) is the second most common variant, representing 9.2% (59/640) and 3.6% (59/1642) of all carriers and participants, respectively. The third most common variant is *G6PD*; c.961G > A (p.Val321Met) causing G6PD deficiency, with 8.6% (55/640) and 3.4% (55/1642) of all carriers and participants, respectively. The variant carrier rates are shown in S3 Table.

### Thalassemia and hemoglobinopathies

Although, the most common type of α-thalassemia mutation is the deletion of one or more of the α-globin genes, *HBA1* and *HBA2* [18], we did not attempt to analyze this type of genetic variant due to the inherent limitation of exome sequencing. The carrier rates of nondeletional α-thalassemia were detected in 5.1% (71/1642; 6 in *HBA1* and 65 in *HBA2*) of our cohort. The most common variant is c.427 T > C 3.6% (59/1642) in Hb Constant Spring, followed by Hb Paksé c.429A > T 0.3% (5/1642). The frequency rate of β-thalassemia carriers is 19.6% (321/1642). The most prevalent variant, c.79G > A (p.Glu27Lys [HbE]), accounts for 17.7% (291/1642) of cases in the HBB genes. The next two most prevalent variants are c.52A > T (0.79%, 13/1642) and c.126_129del (0.67%, 11/1642).

### G6PD deficiency

The carrier rate of G6PD variants was 7.7% (127/1642). The prevalence of heterozygous/carrier G6PD variant and homozygous females in our cohort is 15.28% (127/831) and 0.8% (7/831), respectively (S4 Table). The percentage of symptomatic or biochemical G6PD deficiency in these samples was not known. The prevalence of hemizygous G6PD variant males in our cohort is 8% (65/811). The female carrier rate corresponds with the predicted prevalence calculated with male hemizygous prevalence using the Hardy–Weinberg formula. c.961G > A (p.Val321Met) (G6PD Viantien) and c.577G > A (p.Gly193Ser) (G6PD Mahidol) are the two most common G6PD variants found in our cohort.

### Other autosomal recessive disorder carrier rate and common variants

The carrier frequency of PV and LPV in *GBA* causing Gaucher disease is 1.6% (27/1642) with c.605G > A (p.Arg202Gln) (0.54%, 9/1642) and c.1448 T > C (p.Leu483Pro) (0.42%, 7/1642) being the two most common variants.

The carrier frequency of PV and PLV in genes associated with hereditary syndromic hearing loss *SLC26A2* is 1.1% (18/1642). c.919-2A > G was the most common variant (0.3%, 5/1642). The second most common gene causing syndromic hearing loss carriers was *USH2A* (Usher syndrome) (0.97%, 16/1642). The carrier frequency of PV and PLV in *GJB2*, one of the most common genes causing nonsyndromic deafness is 0.6% (9/1642). All cases identified carried c.235delC (p.Leu79CysfsTer3) in *GJB2*.

Besides Gaucher disease, the most common inherited metabolic disease gene carriers in our cohort are hyperoxaluria (*AGXT*; 1.1%, 18/1642), Wilson disease (*ATP7B*; 1%, 17/1642), and phenylketonuria (*PAH*; 0.79%, 13/1642).

## Discussion

Every approach possesses both advantages and limitations. In our study, we chose to engage parents whose children were afflicted with rare diseases rather than employing a population-based screening methodology. This strategy, akin to "killing two birds with one stone," aimed to optimize the benefits derived from trio exome testing. Specifically, children with rare diseases stood a higher chance of obtaining molecular diagnoses through trio exomes as opposed to singleton testing. Furthermore, the exomes of the parents provided valuable insights into a spectrum of other biological and medical issues. To mitigate bias, we deliberately excluded genes identified as causative for rare diseases in the children. Notably, our approach is characterized by a lower ethical burden. The apparently healthy parents, whose children were affected by rare diseases in our cohort, received genetic counseling and were well-informed about the implications of these tests. They made a conscious choice to permit the use of their genomic information for purposes beyond the primary focus of the testing.Conversely, a population-based approach is not without its constraints. Recent evidence [19, 20], suggests the presence of participation bias. Additionally, ascertainment bias may manifest, with individuals referred or participating in the project having a known family history of rare diseases showing heightened interest. The ethical complexities surrounding population screening pose a challenge. Carrier screening currently remains optional and not a standard mandatory test. While couples undergoing population screening could potentially benefit from counseling, extending genetic counseling to research subjects who are apparently normal may be cumbersome and miss the opportunity to support those genuinely in need. Moreover, the practical medical advantages of counseling may be constrained by the exorbitant costs associated with reproductive choices such as in vitro fertilization (IVF) and preimplantation genetic diagnosis (PGD).

Prior investigations into the carrier rates of these disorders in various countries have exhibited substantial variability in terms of the number of genes tested and the specific genes encompassed within the studies across diverse populations. Notably, within the Thai population, genetic disorders that manifest with a comparatively higher prevalence than in other populations encompass thalassemia, hemoglobinopathies, and glucose-6-phosphate dehydrogenase (G6PD) deficiency [21]. Carriers of these hematological disorders in Thais demonstrate a relative resistance to malaria, affording them a selective advantage. Since HBA and HBB are already incorporated into the ACMG 113-gene list, we have augmented our gene panel to encompass G6PD deficiency, resulting in a total of 114 genes. This expansion aligns with our commitment to comprehensively address the genetic landscape relevant to carrier screening, particularly in the context of the prevalent hematological disorders observed in the Thai population.

Next-Generation Sequencing (NGS) has been increasingly used for expanded genetic carrier screening for multiple genes in a highly efficient manner in clinical laboratories [22]. When screening for carriers in individuals with an average risk, it is deemed acceptable or advisable to report solely (likely) pathogenic variants [13]. The carrier rate ranges from as high as 43.6% in Ashkenazi Jewish individuals to 8.5% in East Asians [23], although the number of genes tested and which genes are included are varied among these studies. In our study using 1642 exomes of a Thai population, 39% of Thai individuals carried a PV and an LPV in at least one of 114 genes (113 genes have been recommended by ACMG [12]) plus *G6PD*, causing G6PD deficiency, which is most common in the Thai population. We observed that 23.8% of individuals were carriers of thalassemia and hemoglobinopathies (*HBB*, *HBA1*, and *HBA2*), followed by 7.7% who were carries of G6PD deficiency. The carrier rate observed in our cohort is considered high, comparable to that of the Ashkenazi Jewish group. The majority of these variants are found in the Hb genes and G6PD, which are believed to be the result of heterozygous advantage in regions with a high prevalence of malaria [24]. This differs from the Ashkenazi Jewish population, where the high carrier rate is primarily attributed to in-group mating.

Consanguineous marriage is generally considered taboo in the majority of the Thai population, with exceptions observed in certain minor communities such as Muslims and hill tribes. Our assertions are substantiated by evidence gleaned from our analysis of inheritance patterns among patients referred for genetic testing at our institution. Notably, our findings reveal that only 25% of positive tests exhibit inheritance in an autosomal recessive pattern, in stark contrast to Middle Eastern countries where the autosomal recessive pattern can reach up to 80%, particularly in regions where consanguinity is commonplace [25]. It is imperative to underscore that thalassemia and hemoglobinopathies are highly prevalent in our region, and the pseudoautosomal dominant pattern observed in our study is reflective of this prevalence rather than indicative of prevailing marriage patterns.

Even after excluding thalassemia and hemoglobinopathies, the carrier rate in our cohort remained significantly high at 14.7%. We observed 14 couples (1.7%) who were carriers of PV and LPV in the same gene (S5 Table). Of these couples, seven (0.9%, 7/821) were non thalassemia carrier couples at risk of having children affected with recessive disorders. Thalassemia carrier screening is already included and available in the screening program of Thai pregnant women; therefore, it is estimated that nearly 1% of the couples could benefit from expanded carrier screening.

The prevalence of common recessive disorders in our cohort can be estimated and, in general, is comparable to previous prevalence rates reported for common autosomal recessive disorders, such as Thalassemia and G6PD deficiency [18, 26–31]. This suggests that our approach of excluding samples carrying genetic variants responsible for the diseases in their offspring during ascertainment and analysis helps eliminate bias and ensures that the frequency estimates are comparable to those of non-disease or population cohorts. We have previously demonstrated the effectiveness of this strategy in our analysis of the prevalence of secondary findings, particularly since the majority of reportable diseases are inherited in an autosomal dominant pattern.

The most common carriers of inherited metabolic disease identified in our cohort are individuals with Gaucher disease caused by mutations in the GBA gene. However, interpreting the results can be challenging due to the presence of a pseudogene associated with GBA. The GBA pseudogene exhibits the highest homology with GBA between exons 8 and 11, which is likely a result of recombination events [32]. Many clinical exome sequencing laboratories do not analyze or report the variants in *GBA*. However, within the exome, there exists a mappable region of *GBA* that encompasses known recurrent pathogenic variants, including the c.1448 T > C mutation. This region can be analyzed with a high degree of sensitivity and specificity. This methodology has been detailed in our recent publication on early-onset Parkinson’s disease in Thai cohorts [33]. In addition, the variants commonly identified in our cohort are outside these highly homologous areas and are therefore less likely to be affected in our analysis. Conversely, the prevalence of the carrier of *GBA* variant is likely to be underestimated in our cohort or a similar cohort using exome sequencing. Nevertheless, it is crucial to acknowledge the limitation associated with utilizing exome sequencing for *GBA* carrier screening. While this approach offers high sensitivity and specificity for certain regions, its effectiveness in reducing the posttest probability of being a *GBA* carrier for other areas is comparatively low. This limitation underscores the importance of considering alternative testing methods for a comprehensive and accurate assessment of *GBA* carrier status.

In our study, carriers of primary hyperoxaluria and Wilson disease PV and PLV were found to be the next most common. Founder variants in East Asian populations (c.2 T > C (p.Met1Thr) in *AGXT* [34–37] and c.2333G > T (p.Arg778Leu) in *ATP7B* [38–43] were also identified in our cohort. Our study further supports the existing newborn screening program in Thailand for Phenylketonuria (PKU). The estimated carrier rate of 1 in 127 individuals is comparable to rates reported in other countries [44–46]. With an annual live birth rate of 600,000, our findings suggest that approximately 8–10 children will be born with PKU each year in Thailand. These findings emphasize the importance of continued implementation and monitoring of the newborn screening program for PKU in the country.

Hearing loss is one of the major disability problems in childhood, with hereditary causes, both syndromic and nonsyndromic, being the primary etiologies in affected children. In our study, we identified carriers of Pendred syndrome, Usher syndrome, and GJB2 (nonsyndromic hereditary neurosensory hearing loss) to be common in the Thai cohort, aligning with previous estimations [47–58]. These findings highlight the relevance of these hereditary conditions as significant contributors to hearing loss in the Thai population.

Compared to the common recommendation of screening for cystic fibrosis carrier status in Caucasians [59–61], our study revealed that only 0.97% of our sample carried pathogenic variants (PV) and likely pathogenic variants (LPV) in the CFTR gene. This suggests that the estimated prevalence of cystic fibrosis among Thais is lower than that among Caucasians (1 in 2,000 in Northern European descent vs. 1 in 400,000). Interestingly, we observed three females (0.18%) who carried the 5T variant (c.1210–12 [5]) in CFTR. While this variant is associated with mild cystic fibrosis, it can result in male infertility due to congenital bilateral absence of the vas deferens [62].

## Limitations

Our study had some limitations. Whole Exome Sequencing (WES) cannot reliably detect structural variants that are common in α-thalassemia. Spinal muscular atrophy, a common muscular disorder, most commonly caused by a copy number change in the *SMN1* gene and modified by the copy numbers of *SMN2*, is also missed by WES. The genome analysis with structural variant detection will help estimate these common disorders. Additionally, the frequency of variants based on NGS analysis may be underestimated without validation using Sanger sequencing, particularly for complex recombinant alleles of GBA pseudogenes. The other common PVs that will be missed by WES are trinucleotide repeat expansion. PV in *FMR1* causing fragile X syndrome, the most common cause of intellectual disability especially in males, cannot be detected with exome sequencing data. It is worth mentioning that our sample size provides over 80% power to detect alleles with a frequency of 1%. However, for the detection of uncommon variants, a larger sample size would be necessary [63].

## Conclusion

Among the 114 genes analyzed, which included the 113 ACMG-recommended genes along with G6PD, our study revealed that 39% (640/1642) of Thai individuals in our cohort were carriers of pathogenic variants (PVs). Through a combination of computational filtering and manual curation, we were able to unveil the landscape of these PVs that are commonly observed in the Thai population. This information will serve as a foundation for designing gene lists for individual-level carrier screening and future public health plans aimed at addressing inherited genetic disorders.

### Supplementary Information

**Additional file 1: S1 Table.** Genetic variants of the 18 parents which were responsible for the presenting symptoms in their children. **S2 Table.** Carrier frequencies of one or more genetic disorders in 114 genes of 1,642 unrelated Thais. **S3 Table.** Variant carrier rate (VCR) of each variant. **S4 Table.** Characteristics of G6PD phenotypes in 1,642 unrelated Thais. **S5 Table.** Pathogenic or likely pathogenic variants in the same genes which were harbored by the couples.

## Data Availability

The datasets used and/or analyzed during the current study available from the corresponding author on reasonable request.

## Abbreviations

ACMG: The American College of Medical Genetics and Genomics
LPV: Likely pathogenic variants
PV: Pathogenic variants
NGS: Next-Generation Sequencing
WES: Whole exome sequencing
