# Supplementary material for: Carrier frequency estimation of pathogenic variants of autosomal recessive and X-linked recessive mendelian disorders using exome sequencing data in 1,642 Thais
Source: BMC Med Genomics. 2024 Jan 2;17:9. doi: 10.1186/s12920-023-01771-w (PMC10762924; doi:10.1186/s12920-023-01771-w)
Supplement: Supplementary file 1 — Additional file 1: S1 Table. Genetic variants of the 18 parents which were responsible for the presenting symptoms in their children. S2 Table. Carrier frequencies of one or more genetic disorders in 114 genes of 1,642 unrelated Thais. S3 Table. Variant carrier rate (VCR) of each variant. S4 Table. Characteristics of G6PD phenotypes in 1,642 unrelated Thais. S5 Table. Pathogenic or likely pathogenic variants in the same genes which were harbored by the couples. [file 12920_2023_1771_MOESM1_ESM.docx]

**S1 Table.** Genetic variants of the 18 parents which were responsible for the presenting symptoms in their children

|  | HGVS | RSID | Family ID | Proband' disease | Inherited from |
| --- | --- | --- | --- | --- | --- |
| *ATP7B* (4) | c.1708-1G>C | rs137853280 | FAMA | Wilson diseases | Father |
|  | c.3426G>C (p.Gln1142His) | rs778749563 |  |  | Mother |
|  | c.2333G>T (p.Arg778Leu) | rs28942074 | FAMB | Wilson diseases | Father |
|  | c.2605G>A (p.Gly869Arg) | rs191312027 |  |  | Mother |
| *BCKDHB* (1) | c.403G>A (p.Gly135Arg) | rs137853280 | FAMC | Maple syrup urine disease | Father |
| *CFTR* (4) | c.2909G>A (p.Gly970Asp) | rs386134230 | FAMD | Cystic fibrosis | Father |
|  | c.2834C>T (p.Ser945Leu) | rs397508442 |  |  | Mother |
|  | c.3883_3886del (p.Ile1295PhefsTer32) | rs387906373 | FAME | Recurrent pneumonia , Failure future | Father |
|  | c.3883_3886del (p.Ile1295PhefsTer32) | rs387906373 |  |  | Mother |
| *HBB* (2) | c.217dup (p.Ser73LysfsTer2) | rs33969853 | FAMF | Beta-thalassemia trait | Mother |
|  | c.92+1G>T | rs33971440 |  |  | Father |
| *HBA2* (5) | c.427T>C (p.Ter143Glnext*31) | rs41464951 | FAMG | Hemolytic anemia | Mother |
|  | c.427T>C (p.Ter143Glnext*31) | rs41464951 | FAMH | Severe fetal anemia HPP | Father |
|  | c.427T>C (p.Ter143Glnext*31) | rs41464951 |  |  | Mother |
|  | c.427T>C (p.Ter143Glnext*31) | rs41464951 | FAMI | Alpha-thalassemia | Father |
|  | c.427T>C (p.Ter143Glnext*31) | rs41464951 |  |  | Mother |
| *NEB* (2) | c.9883C>T (p.Arg3295Ter) | rs779909544 | FAMJ | Congenital myopathies | Father |
|  | c.24072_24075del (p.Pro8025SerfsTer154) | rs756384471 |  |  | Mother |

**S2 Table.** Carrier frequencies of one or more genetic disorders in 114 genes of 1,642 unrelated Thais

|  | Carrier frequency of  1 disorder  [≥ 1 disorders] | Carrier frequency of  2 disorders  [≥ 2 disorders] | Carrier frequency of  3 disorders  [≥ 3 disorders] | Carrier frequency of  4 disorders |
| --- | --- | --- | --- | --- |
| 110 genes  (the 113 ACMG-recommended genes excluding thalassemia and hemoglobinopathies:  *HBB*, *HBA1*, and *HBA2*) | 13.34% (219/1642)  [frequency of ≥ 1 disorder  = 14.7%  (13.34+1.22+0.12)] | 1.22% (20/1642)  [frequency of ≥ 2 disorders  = 1.3%  (1.22+0.12)] | 0.12% (2/1642) | 0 |
| The 113 ACMG-recommended genes | 28.26% (464/1642)  [frequency of ≥ 1 disorder  = 34.0%  (28.26+5.12+0.43+0.06)] | 5.12% (84/1642)  [frequency of ≥ 2 disorders   = 5.6%  (5.12+0.43+0.06)] | 0.43% (7/1642)  [frequency of ≥ 3 disorders  = 0.5%  (0.43+0.06)] | 0.06% (1/1642) |
| 114 genes  (the 113 ACMG-recommended genes + G6PD) | 31.73% (521/1642)  [frequency of ≥ 1 disorder  = 39.1%  (31.73+6.33+0.85+0.18)] | 6.33% (104/1642)  [frequency of ≥ 2 disorders  = 7.4%  (6.33+0.85+0.18)] | 0.85% (14/1642)  [frequency of ≥ 3 disorders  = 1%  (0.85+0.18)] | 0.18% (3/1642) |

**S3 Table.** Variant carrier rate (VCR) of each variant

| Gene | HGVS | RSID | Number  of  individual | VCR(%) | 1 in | Varsome classification |
| --- | --- | --- | --- | --- | --- | --- |
| *ABCC8*(2) | c.3000C>A (p.Cys1000Ter) | rs192863214 | 1 | 0.06 (1/1642) | 1642 | P;PVS1,PM2,PP5 |
|  | c.2797C>T (p.Arg933Ter) | rs570388861 | 1 | 0.06 (1/1642) | 1642 | P;PVS1,PP5,PM2 |
| *ABCD1*(5) | c.1699C>T (p.Gln567Ter) | rs201114595 | 5 | 0.30 (5/1642) | 329 | P;PVS1,PP5 |
| *ACADM*(1) | c.461_464del (p.Thr154ArgfsTer4) | rs786204642 | 1 | 0.06 (1/1642) | 1642 | P;PVS1,PP5,PM2 |
| *ACAT1*(1) | c.622C>T (p.Arg208Ter) | rs532190594 | 1 | 0.06 (1/1642) | 1642 | P;PVS1,PP5,PM2 |
| *AGXT*(18) | c.2T>C (p.Met1Thr) | rs138584408 | 12 | 0.73 (12/1642) | 137 | P;PVS1,PP5,PM2 |
|  | c.32C>G (p.Pro11Arg) | rs34116584 | 4 | 0.24 (4/1642) | 411 | P;PP5,PM2,PP3 |
|  | c.466G>A (p.Gly156Arg) | rs121908530 | 1 | 0.06 (1/1642) | 1642 | P;PS1,PS3,PM1,PM2,PM5,PP3 |
|  | c.1079G>A (p.Arg360Gln) | rs180177161 | 1 | 0.06 (1/1642) | 1642 | P;PM1,PM2,PM5,PP3,PP5 |
| *AIRE*(5) | c.652+1G>T | rs199612115 | 5 | 0.30 (5/1642) | 329 | P;PVS1,PP5,PM2,PS3 |
| *ALDOB*(1) | c.1013C>T (p.Ala338Val) | rs77718928 | 1 | 0.06 (1/1642) | 1642 | P;PP5,PM2,PP2,PP3 |
| *ALPL*(3) | c.211C>T (p.Arg71Cys) | rs121918001 | 1 | 0.06 (1/1642) | 1642 | P;PM1,PM2,PM5,PP5,PP3 |
|  | c.542C>T (p.Ser181Leu) | rs199590449 | 1 | 0.06 (1/1642) | 1642 | P;PP5,PM1,PM5,PM2,PP3 |
|  | c.979T>C (p.Phe327Leu) | rs121918010 | 1 | 0.06 (1/1642) | 1642 | P;PP5,PS1,PM1,PM2,PM5,PP3 |
| *ARSA*(2) | c.736C>T (p.Arg246Cys) | rs74315470 | 1 | 0.06 (1/1642) | 1642 | P;PP5,PM1,PM2,PM5,PP3 |
|  | c.2T>C (p.Met1Thr) | rs1555901170 | 1 | 0.06 (1/1642) | 1642 | P;PVS1,PM2,PP5 |
| *ATP7B*(17) | c.3646G>A (p.Val1216Met) | rs776280797 | 1 | 0.06 (1/1642) | 1642 | P;PP5,PM1,PM2,PP3 |
|  | c.3426G>C (p.Gln1142His) | rs778749563 | 1 | 0.06 (1/1642) | 1642 | LP;PS1,PM1,PM2 |
|  | c.3316G>A (p.Val1106Ile) | rs541208827 | 3 | 0.18 (3/1642) | 548 | LP;PM1,PM2,PM5,PP5 |
|  | c.2975C>T (p.Pro992Leu) | rs201038679 | 1 | 0.06 (1/1642) | 1642 | P;PP5,PM1,PM2,PM5,PP3 |
|  | c.2804C>T (p.Thr935Met) | rs750019452 | 1 | 0.06 (1/1642) | 1642 | P;PP5,PM1,PM2,PP3 |
|  | c.2755C>G (p.Arg919Gly) | rs121907993 | 3 | 0.18 (3/1642) | 548 | P;PP5,PM1,PM2,PM5,PP3 |
|  | c.2692C>T (p.Gln898Ter) | . | 1 | 0.06 (1/1642) | 1642 | P;PVS1,PM2,PP5,PP3 |
|  | c.2605G>A (p.Gly869Arg) | rs191312027 | 1 | 0.06 (1/1642) | 1642 | P;PM1,PM2,PM5,PP5 |
|  | c.2333G>T (p.Arg778Leu) | rs28942074 | 2 | 0.12 (2/1642) | 821 | P;PP5,PM1,PM2,PM5,PS3,PP3 |
|  | c.2128G>A (p.Gly710Ser) | rs137853285 | 1 | 0.06 (1/1642) | 1642 | P;PP5,PM1,PM2,PM5,PP3 |
|  | c.813C>A (p.Cys271Ter) | rs572147914 | 1 | 0.06 (1/1642) | 1642 | P;PVS1,PP5,PM2 |
|  | c.314C>A (p.Ser105Ter) | rs753236073 | 1 | 0.06 (1/1642) | 1642 | P;PVS1,PP5,PM2 |
| *BBS2*(3) | c.2107C>T (p.Arg703Ter) | rs567573386 | 2 | 0.12 (2/1642) | 821 | P;PVS1,PP5,PM2 |
|  | c.700C>T (p.Arg234Ter) | rs779690256 | 1 | 0.06 (1/1642) | 1642 | P;PVS1,PP5,PM2 |
| *BTD*(1) | c.571C>T (p.Arg191Cys) | rs372844636 | 1 | 0.06 (1/1642) | 1642 | P;PP5,PM1,PM2,PP3 |
| *CBS(*2) | c.1058C>T (p.Thr353Met) | rs121964972 | 1 | 0.06 (1/1642) | 1642 | P;PS3,PP5,PM1,PM2,PP3 |
|  | c.833T>C (p.Ile278Thr) | rs5742905 | 1 | 0.06 (1/1642) | 1642 | P;PM2,PM5,PP5,PP2 |
| *CC2D2A*(2) | c.4465_4468del (p.Asp1489LysfsTer15) | rs797045437 | 2 | 0.12 (2/1642) | 821 | P;PVS1,PP5,PM2 |
| *CEP290*(7) | c.6869del (p.Asn2290IlefsTer11) | rs587783017 | 2 | 0.12 (2/1642) | 821 | P;PVS1,PM2,PP5 |
|  | c.6798G>A (p.Trp2266Ter) | rs760540562 | 1 | 0.06 (1/1642) | 1642 | P;PVS1,PM2,PP5 |
|  | c.5668G>T (p.Gly1890Ter) | rs137852832 | 1 | 0.06 (1/1642) | 1642 | P;PVS1,PP5,PM2 |
|  | c.4962_4963del (p.Glu1656AsnfsTer3) | rs764309755 | 1 | 0.06 (1/1642) | 1642 | P;PVS1,PP5,PM2 |
|  | c.4813-2A>G | rs369523378 | 1 | 0.06 (1/1642) | 1642 | P;PVS1,PP5,PM2 |
|  | c.3814C>T (p.Arg1272Ter) | rs62640581 | 1 | 0.06 (1/1642) | 1642 | P;PVS1,PP5,PM2 |
| *CFTR*(16) | c.263T>A (p.Leu88Ter) | rs397508412 | 1 | 0.06 (1/1642) | 1642 | P;PVS1,PP5,PM2 |
|  | c.273+1G>A | rs121908791 | 1 | 0.06 (1/1642) | 1642 | P;PVS1,PP5,PM2 |
|  | c.349C>T (p.Arg117Cys) | rs77834169 | 2 | 0.12 (2/1642) | 821 | P;PP5,PM1,PM2,PM5,PP3 |
|  | c.1234_1238del (p.Ala412ThrfsTer4) | rs3034796 | 5 | 0.30 (5/1642) | 329 | P;PVS1,PP5,PM2 |
|  | c.1657C>G (p.Arg553Gly) | rs74597325 | 1 | 0.06 (1/1642) | 1642 | P;PM1,PM5,PM2,PP3 |
|  | c.1865G>A (p.Gly622Asp) | rs121908759 | 2 | 0.12 (2/1642) | 821 | LP;PM1,PM2,PP3,PP5 |
|  | c.2845C>T (p.His949Tyr) | rs121909035 | 1 | 0.06 (1/1642) | 1642 | LP;PM1,PM2,PP5,PP3 |
|  | c.3472C>T (p.Arg1158Ter) | rs79850223 | 1 | 0.06 (1/1642) | 1642 | P;PVS1,PP5,PM2 |
|  | c.3883_3886del (p.Ile1295PhefsTer32) | rs387906373 | 2 | 0.12 (2/1642) | 821 | P;PVS1,PP5,PM2 |
| *CLCN1*(7) | c.871G>A (p.Glu291Lys) | rs121912805 | 2 | 0.12 (2/1642) | 821 | P;PM2,PM1,PP5,PP3 |
|  | c.1663C>T (p.His555Tyr) | rs201850090 | 5 | 0.30 (5/1642) | 329 | LP;PM1,PM2,PP3,PP5 |
| *CPT2*(1) | c.1148T>A (p.Phe383Tyr) | rs74315295 | 1 | 0.06 (1/1642) | 1642 | P;PP5,PM2 |
| *CYP27A1*(7) | c.379C>G (p.Arg127Gly) | rs201114717 | 1 | 0.06 (1/1642) | 1642 | P;PM2,PM5,PP3,PP5 |
|  | c.1415G>C (p.Gly472Ala) | rs200883871 | 6 | 0.37 (6/1642) | 274 | LP;PM1,PM2,PP3,PP5 |
| *DHCR7*(2) | c.725G>A (p.Arg242His) | rs80338857 | 2 | 0.12 (2/1642) | 821 | P;PP5,PM1,PM2,PM5,PP3 |
| *DLD*(2) | c.140T>C (p.Ile47Thr) | rs397514651 | 2 | 0.12 (2/1642) | 821 | LP;PM2,PP5,PP3 |
| *DYNC2H1*(2) | c.988C>T (p.Arg330Cys) | rs397514637 | 1 | 0.06 (1/1642) | 1642 | P,PM2,PP5,PP3,PM5 |
|  | c.7967G>T (p.Arg2656Leu) | rs200614421 | 1 | 0.06 (1/1642) | 1642 | P;PM1,PM2,PM5,PP5 |
| *ERCC2*(1) | c.2092C>T (p.Gln698Ter) | rs1555775416 | 1 | 0.06 (1/1642) | 1642 | P;PVS1,PM2,PP5 |
| *EVC2*(3) | c.2029C>T (p.Arg677Ter) | rs73198165 | 1 | 0.06 (1/1642) | 1642 | P;PVS1,PM2,PP5 |
|  | c.1195C>T (p.Arg399Ter) | rs137852924 | 2 | 0.12 (2/1642) | 821 | P;PVS1,PP5,PM2 |
| *FAH*(2) | c.709C>T (p.Arg237Ter) | rs769550316 | 1 | 0.06 (1/1642) | 1642 | P;PVS1,PP5,PM2 |
|  | c.782C>T (p.Pro261Leu) | rs80338898 | 1 | 0.06 (1/1642) | 1642 | P;PP5,PM2,PP3 |
| *FANCC*(1) | c.1257del (p.Thr420ArgfsTer27) | rs765551897 | 1 | 0.06 (1/1642) | 1642 | P;PVS1,PM2,PP5 |
| *FKRP*(2) | c.545A>G (p.Tyr182Cys) | rs543163491 | 2 | 0.12 (2/1642) | 821 | P;PP5,PM1,PM2,PM5 |
| *FKTN*(2) | c.919C>T (p.Arg307Ter) | rs267606814 | 1 | 0.06 (1/1642) | 1642 | LP;PP3,PP5,PM2,PM5 |
|  | c.1106del (p.Phe369SerfsTer37) | rs750176716 | 1 | 0.06 (1/1642) | 1642 | P;PVS1,PP5,PM2 |
| *FMO3*(2) | c.591_592del (p.Cys197Ter) | rs3832024 | 2 | 0.12 (2/1642) | 821 | P;PVS1,PM2,PP5 |
|  | c.1160G>T (p.Arg387Leu) | rs72549331 | 1 | 0.06 (1/1642) | 1642 | LP;PM2,PM5,PP3,PP5 |
| *G6PD*(127) | c.961G>A (p.Val321Met) | rs137852327 | 55 | 3.35 (55/1642) | 79 | P;PP5,PS3,PP2 |
|  | c.577G>A (p.Gly193Ser) | rs137852314 | 33 | 2.0 (33/1642) | 79 | P;PP5,PM1,PM5,,PP3 |
|  | c.1478G>A (p.Arg493His) | rs72554664 | 14 | 0.85 (14/1642) | 411 | P;PP5,PM1,PM5,BP4,BP6 |
|  | c.1466G>T (p.Arg489Leu) | rs72554665 | 11 | 0.67 (11/1642) | 411 | P;PP5,PM1,PM5,PS3,BP4 |
|  | c.1450C>T (p.Arg484Cys) | rs398123546 | 9 | 0.55 (9/1642) | 206 | P;PP5,PM5,PM1,PP3 |
|  | c.221C>G (p.Ala74Gly) | rs78478128 | 3 | 0.18 (3/1642) | 821 | P;PP5,PM1,PM5 |
|  | c.653C>T (p.Ser218Phe) | rs5030868 | 2 | 0.12 (2/1642) | 1642 | P;PP5,PS3,PM1,BP4 |
| *GAA*(4) | c.546G>A (p.Thr182=) | rs143523371 | 1 | 0.06 (1/1642) | 1642 | P;PP3,PP5,PM2 |
|  | c.784G>A (p.Glu262Lys) | rs201896815 | 1 | 0.06 (1/1642) | 1642 | P;PP5,PM2,PP3,PM1 |
|  | c.1843G>A (p.Gly615Arg) | rs549029029 | 1 | 0.06 (1/1642) | 1642 | P;PS1,PM1,PP5,PM2,PM5,PP3 |
|  | c.2662G>T (p.Glu888Ter) | rs765718882 | 1 | 0.06 (1/1642) | 1642 | P;PVS1,PP5,PM2 |
| *GALT*(2) | c.428C>A (p.Ser143Ter) | rs111033697 | 1 | 0.06 (1/1642) | 1642 | LP;PVS1,PM2 |
|  | c.998G>A (p.Arg333Gln) | rs111033808 | 1 | 0.06 (1/1642) | 1642 | P;PP5,PM1,PM2,PM5,PP3,PS3 |
| *GBA*(27) | c.1609T>C (p.Ter537Argext*15) | . | 2 | 0.12 (2/1642) | 821 | LP;PM4,PM2,BP4 |
|  | c.1483G>C (p.Ala495Pro) | rs368060 | 1 | 0.06 (1/1642) | 1642 | LP;PS3,PM1,PM2,PP5 |
|  | c.1448T>C (p.Leu483Pro) | rs421016 | 7 | 0.43 (7/1642) | 235 | P;PS3,PM1,PM5,PP5,PM2,PP3 |
|  | c.1224G>A (p.Thr408=) | rs138498426 | 1 | 0.06 (1/1642) | 1642 | LP;PP3,PM2 VUS: |
|  | c.1223C>T (p.Thr408Met) | rs75548401 | 1 | 0.06 (1/1642) | 1642 | LP;PM1,PP2,PP5,BS2 : LB |
|  | c.928A>G (p.Ser310Gly) | rs1057942 | 1 | 0.06 (1/1642) | 1642 | LP;PM1,PP5,PM2,PP3 |
|  | c.762-1G>C | . | 1 | 0.06 (1/1642) | 1642 | P;PVS1,PM2,PP5 |
|  | c.605G>A (p.Arg202Gln) | rs398123531 | 9 | 0.55 (9/1642) | 183 | LP;PM1,PM2,PP2 LB |
|  | c.586A>C (p.Lys196Gln) | rs121908297 | 3 | 0.18 (3/1642) | 548 | LP;PS3,PM2,PP2,PP3 |
|  | c.204_205insC (p.Thr69HisfsTer12) | . | 1 | 0.06 (1/1642) | 1642 | LP;PVS1,PM2 |
| *GBE1*(2) | c.1561A>T (p.Lys521Ter) | rs773775991 | 1 | 0.06 (1/1642) | 1642 | P;PVS1,PM2,PP5 |
|  | c.1300C>T (p.Arg434Ter) | rs781198373 | 1 | 0.06 (1/1642) | 1642 | P;PVS1,PM2,PP5 |
| *GJB2*(9) | c.235del (p.Leu79CysfsTer3) | rs80338943 | 9 | 0.55 (9/1642) | 183 | P;PVS1,PS3,PP5,PM2 |
| *GNPTAB9*(1) | c.2550_2554del (p.Lys850AsnfsTer10) | rs281864996 | 1 | 0.06 (1/1642) | 1642 | P;PVS1,PP5,PM2 |
| *HBA1*(5) | c.1del (p.Met1TrpfsTer49) | . | 3 | 0.18 (3/1642) | 548 | LP;PVS1,PM2 |
|  | c.95+1G>A | rs1201093320 | 2 | 0.12 (2/1642) | 821 | P;PVS1,PM2,PP5 |
| *HBA2*(65) | c.377T>C (p.Leu126Pro) | rs41397847 | 1 | 0.06 (1/1642) | 1642 | P;PM1,PM5,PM2,PP3,PP5 |
|  | c.427T>C (p.Ter143Glnext*31) | rs41464951 | 59 | 3.59 (59/1642) | 28 | P;PP5,PM2,PM4,BP4 |
|  | c.429A>T (p.Ter143Tyrext*31) | rs41412046 | 5 | 0.30 (5/1642) | 329 | P;PP5,PM2,PM4,BP4 |
| *HBB*(321) | c.217dup (p.Ser73LysfsTer2) | rs33969853 | 1 | 0.06 (1/1642) | 1642 | P;PVS1,PS3,PM2,PP5 |
|  | c.126_129del (p.Phe42LeufsTer19) | rs80356821 | 11 | 0.67 (11/1642) | 150 | P;PVS1,PP5,PM2 |
|  | c.108C>A (p.Tyr36Ter) | rs33982568 | 1 | 0.06 (1/1642) | 1642 | P;PVS1,PM2,PP5 |
|  | c.92+5G>C | rs33915217 | 1 | 0.06 (1/1642) | 1642 | P;PS3,PP5,PP3,PM2 |
|  | c.92+1G>T | rs33971440 | 3 | 0.18 (3/1642) | 548 | P;PVS1,PP5,PM2 |
|  | c.52A>T (p.Lys18Ter) | rs33986703 | 13 | 0.79 (13/1642) | 127 | P;PVS1,PP5,PM2 |
|  | c.79G>A (p.Glu27Lys) | rs33950507 | 291 | 17.72 (291/1642) | 6 | P;PS3,PP5,PM2,PM1,PM5,BP4 |
| *HEXA*(1) | c.508C>T (p.Arg170Trp) | rs121907972 | 1 | 0.06 (1/1642) | 1642 | P;PP5,PM1,PM2,PM5,PP3 |
| *HPS1*(1) | c.973_974insC (p.Met325ThrfsTer128) | . | 1 | 0.06 (1/1642) | 1642 | LP;PVS1,PM2 |
| *IDUA*(1) | c.1898C>T (p.Ser633Leu) | rs886043347 | 1 | 0.06 (1/1642) | 1642 | P;PP5,PM1,PM2,PM5,PP3 |
| *MCCC2*(1) | c.1015G>A (p.Val339Met) | rs150591260 | 1 | 0.06 (1/1642) | 1642 | P;PP5,PS3,PM1,PM2 |
| *MCPH1*(8) | c.313A>T (p.Lys105Ter) | rs1424203921 | 1 | 0.06 (1/1642) | 1642 | P;PVS1,PM2,PP5 |
|  | c.1974-2A>G | rs541042265 | 7 | 0.43 (7/1642) | 235 | P;PVS1,PM2,PP5 |
| *MMACHC*(3) | c.217C>T (p.Arg73Ter) | rs796051995 | 1 | 0.06 (1/1642) | 1642 | P;PVS1,PP5,PM2 |
|  | c.609G>A (p.Trp203Ter) | rs587776889 | 1 | 0.06 (1/1642) | 1642 | P;PVS1,PP5,PM2 |
|  | c.666C>A (p.Tyr222Ter) | rs201266016 | 1 | 0.06 (1/1642) | 1642 | P;PVS1,PP5,PM2 |
| *MMUT*(3) | c.1207C>T (p.Arg403Ter) | rs727504020 | 1 | 0.06 (1/1642) | 1642 | P;PVS1,PP5,PM2 |
|  | c.1038_1040del (p.Leu347del) | rs765373403 | 1 | 0.06 (1/1642) | 1642 | P;PP5,PM2,PM1,PP3 |
| *NEB*(12) | c.19142_19163del (p.Thr6381ArgfsTer36) | rs761067911 | 7 | 0.43 (7/1642) | 235 | P;PVS1,PM2,PP5 |
|  | c.24094C>T (p.Arg8032Ter) | rs549794342 | 1 | 0.06 (1/1642) | 1642 | P;PVS1,PP5,PM2 |
|  | c.15364-1G>T | rs760082702 | 2 | 0.12 (2/1642) | 821 | P;PVS1,PM2,PP5 |
|  | c.6183+1G>A | rs557870969 | 1 | 0.06 (1/1642) | 1642 | P;PVS1,PM2,PP5 |
|  | c.1152+1G>A | rs398124167 | 1 | 0.06 (1/1642) | 1642 | P;PVS1,PP5,PM2 |
| *NPHS1*(1) | c.1707C>G (p.Ser569Arg) | rs386833888 | 1 | 0.06 (1/1642) | 1642 | P,PS1,PM1,PM2,PP5,PP3 |
| *OTC*(1) | c.386G>A (p.Arg129His) | rs66656800 | 1 | 0.06 (1/1642) | 1642 | P;PP5,PM1,PM2,PM5,PP3 |
| *PAH*(13) | c.1315+6T>A | rs62508650 | 1 | 0.06 (1/1642) | 1642 | LP;PP5,PM2,BP4 |
|  | c.1223G>A (p.Arg408Gln) | rs5030859 | 4 | 0.24 (4/1642) | 410.5 | P;PP5,PM1,PM2,PM5,PP3 |
|  | c.842+1G>T | rs5030852 | 1 | 0.06 (1/1642) | 1642 | P;PVS1,PP5,PM2 |
|  | c.722del (p.Arg241ProfsTer100) | rs199475657 | 1 | 0.06 (1/1642) | 1642 | P;PVS1,PP5,PM2 |
|  | c.721C>T (p.Arg241Cys) | rs76687508 | 2 | 0.12 (2/1642) | 821 | P;PP5,PS3,PM1,PM2,PM5 |
|  | c.611A>G (p.Tyr204Cys) | rs62514927 | 1 | 0.06 (1/1642) | 1642 | P;PP5,PS3,PM1,PM2,PP3 |
|  | c.355C>T (p.Pro119Ser) | rs398123292 | 1 | 0.06 (1/1642) | 1642 | P;PP5,PM1,PM2,BP4 |
|  | c.331C>T (p.Arg111Ter) | rs76296470 | 1 | 0.06 (1/1642) | 1642 | P;PVS1,PP5,PM2 |
|  | c.320A>G (p.His107Arg) | rs542645236 | 1 | 0.06 (1/1642) | 1642 | P;PP5,PM1,PM2,PM5,BP4 |
| *PCDH15*(1) | c.1997+1G>T | rs763797356 | 1 | 0.06 (1/1642) | 1642 | P;PVS1,PP5,PM2 |
| *PKHD1*(7) | c.11785+1G>T | . | 1 | 0.06 (1/1642) | 1642 | P;PVS1,PM2,PP3,PP5 |
|  | c.11314C>T (p.Arg3772Ter) | rs199839578 | 1 | 0.06 (1/1642) | 1642 | P;PVS1,PP5,PM2 |
|  | c.10444C>T (p.Arg3482Cys) | rs148617572 | 1 | 0.06 (1/1642) | 1642 | P;PP5,PM2,PP3,PS3,BP1 |
|  | c.9901G>T (p.Glu3301Ter) | rs757099749 | 1 | 0.06 (1/1642) | 1642 | P;PVS1,PM2,PP5 |
|  | c.2341C>T (p.Arg781Ter) | rs398124478 | 2 | 0.12 (2/1642) | 1642 | P;PVS1,PP5,PM2 |
|  | c.1480C>T (p.Arg494Ter) | rs754392766 | 1 | 0.06 (1/1642) | 1642 | P;PVS1,PP5,PM2,PS3 |
| *PMM2*(4) | c.385G>A (p.Val129Met) | rs104894525 | 1 | 0.06 (1/1642) | 1642 | P;PP5,PM1,PM2,PM5,PP3 |
|  | c.421C>T (p.Arg141Cys) | rs746610168 | 1 | 0.06 (1/1642) | 1642 | P;PP5,PM1,PM2,PM5,PP3 |
|  | c.430T>C (p.Phe144Leu) | rs150719105 | 1 | 0.06 (1/1642) | 1642 | P;PP5,PS1,PM1,PM2,PM5,BP4 |
|  | c.470T>C (p.Phe157Ser) | rs190521996 | 1 | 0.06 (1/1642) | 1642 | P;PP5,PM1,PM2,PM5,PP3 |
| *POLG*(2) | c.3470A>G (p.Asn1157Ser) | rs548076633 | 1 | 0.06 (1/1642) | 1642 | P;PM2,PP5,PP3 |
|  | c.3286C>T (p.Arg1096Cys) | rs201732356 | 1 | 0.06 (1/1642) | 1642 | P;PP5,PM2,PM5,PM1,PP3 |
| *PRF1*(1) | c.1349C>T (p.Thr450Met) | rs189650890 | 1 | 0.06 (1/1642) | 1642 | P;PP5,PM5,PM1,PM2,PP3 |
| *RARS2*(1) | c.1A>G (p.Met1Val) | rs774923951 | 1 | 0.06 (1/1642) | 1642 | P;PVS1,PP5,PM2 |
| *SLC26A4*(18) | c.349del (p.Leu117SerfsTer9) | rs1275009555 | 1 | 0.06 (1/1642) | 1642 | P;PVS1,PP5,PM2 |
|  | c.706C>G (p.Leu236Val) | rs111033242 | 2 | 0.12 (2/1642) | 821 | P;PP5,PM1,PM2,PM5 |
|  | c.919-2A>G | rs111033313 | 5 | 0.30 (5/1642) | 329 | P;PVS1,PP5,PS3,PM2 |
|  | c.1003T>C(p.Phe335Leu) | rs111033212 | 2 | 0.12 (2/1642) | 821 | P;PP5,PM2,PM5,BP4 |
|  | c.1079C>T (p.Ala360Val) | rs786204474 | 2 | 0.12 (2/1642) | 821 | P;PP5,PM2,PP3 |
|  | c.1229C>T (p.Thr410Met) | rs111033220 | 2 | 0.12 (2/1642) | 821 | P;PP5,PS3,PM1,PM2,PM5,PP3 |
|  | c.1343C>T (p.Ser448Leu) | rs747076316 | 1 | 0.06 (1/1642) | 1642 | P;PP5,PM2,PM5,PP3 |
|  | c.2086C>T (p.Gln696Ter) | rs752807925 | 2 | 0.12 (2/1642) | 821 | P;PVS1,PP5,PM2 |
|  | c.2168A>G (p.His723Arg) | rs121908362 | 1 | 0.06 (1/1642) | 1642 | P;PP5,PM1,PS3,PM2,PM5 |
| *TYR*(4) | c.346C>T (p.Arg116Ter) | rs61753256 | 1 | 0.06 (1/1642) | 1642 | P;PVS1,PP5,PM2 |
|  | c.896G>A (p.Arg299His) | rs61754375 | 1 | 0.06 (1/1642) | 1642 | P;PP5,PS3,PM1,PM2,PM5,PP3 |
|  | c.1146C>A (p.Asn382Lys) | rs104894315 | 1 | 0.06 (1/1642) | 1642 | P;PS1,PM1,PM2,PP3,PP5 |
|  | c.1209G>T (p.Arg403Ser) | rs104894316 | 1 | 0.06 (1/1642) | 1642 | P;PP5,PS1,PM1,PM2,PP3 |
| *USH2A*(16) | c.15412C>T (p.Gln5138Ter) | rs763463859 | 1 | 0.06 (1/1642) | 1642 | P;PVS1,PM2,PP5 |
|  | c.13010C>T (p.Thr4337Met) | rs527236137 | 1 | 0.06 (1/1642) | 1642 | P;PP5,PM2,PP3,PM1,PM5 |
|  | c.11389+1G>A | rs368770647 | 2 | 0.12 (2/1642) | 821 | P;PVS1,PP5 |
|  | c.9570+1G>A | rs760225886 | 1 | 0.06 (1/1642) | 1642 | P;PVS1,PP5,PM2 |
|  | c.8271T>G (p.Tyr2757Ter) | rs1571774019 | 2 | 0.12 (2/1642) | 1642 | P;PVS1,PM2,PP5 |
|  | c.5572+1G>A | rs775293551 | 2 | 0.12 (2/1642) | 821 | P;PVS1,PP5,PM2 |
|  | c.3327C>A (p.Tyr1109Ter) | rs758705873 | 1 | 0.06 (1/1642) | 1642 | P;PVS1,PM2,PP5 |
|  | c.2802T>G (p.Cys934Trp) | rs201527662 | 5 | 0.30 (5/1642) | 329 | LP;PP5,PM5,PM1,PM2 |
|  | c.187C>T (p.Arg63Ter) | rs781223647 | 1 | 0.06 (1/1642) | 1642 | P;PVS1,PP5,PM2 |
| *XPC*(1) | c.1243C>T (p.Arg415Ter) | rs757958943 | 1 | 0.06 (1/1642) | 1642 | P;PVS1,PP5,PM2 |

**S4 Table.** Characteristics of G6PD phenotypes in 1,642 unrelated Thais

| G6PD phenotypes | Male | Female |
| --- | --- | --- |
| G6PD wild-type | 746 | 697 |
| G6PD carrier | - | 127 |
| G6PD deficiency | 65 | 7 |

**S5 Table.** Pathogenic or likely pathogenic variants in the same genes which were harbored by the couples

| Genes | HGVS | RSID | FamilyID | Parents |
| --- | --- | --- | --- | --- |
| *AGXT*(1Couple) | c.2T>C (p.Met1Thr) | rs138584408 | FAM1 | Father |
|  | c.2T>C (p.Met1Thr) | rs138584408 | FAM1 | Mother |
| *CFTR*(1Couple) | c.3472C>T (p.Arg1158Ter) | rs79850223 | FAM2 | Father |
|  | c.1234_1238del (p.Ala412ThrfsTer4) | rs3034796 | FAM2 | Mother |
| *FAH*(1Couple) | c.709C>T (p.Arg237Ter) | rs769550316 | FAM4 | Father |
|  | c.782C>T (p.Pro261Leu) | rs80338898 | FAM4 | Mother |
| *GBA*(1Couple) | c.1448T>C (p.Leu483Pro) | rs421016 | FAM5 | Father |
|  | c.1448T>C (p.Leu483Pro) | rs421016 | FAM5 | Mother |
| *HBA2*(1Couple) | c.427T>C (p.Ter143Glnext*31) | rs41464951 | FAM6 | Father |
|  | c.427T>C (p.Ter143Glnext*31) | rs41464951 | FAM6 | Mother |
| *HBB*(7Couple) | c.126_129del (p.Phe42LeufsTer19) | rs80356821 | FAM8 | Father |
|  | c.92+1G>T | rs33971440 | FAM8 | Mother |
|  | c.79G>A (p.Glu27Lys) | rs33950507 | FAM13 | Father |
|  | c.52A>T (p.Lys18Ter) | rs33986703 | FAM13 | Mother |
|  | c.52A>T (p.Lys18Ter) | rs33986703 | FAM19 | Father |
|  | c.79G>A (p.Glu27Lys) | rs33950507 | FAM19 | Mother |
|  | c.52A>T (p.Lys18Ter) | rs33986703 | FAM21 | Father |
|  | c.79G>A (p.Glu27Lys) | rs33950507 | FAM21 | Mother |
|  | c.79G>A (p.Glu27Lys) | rs33950507 | FAM26 | Father |
|  | c.217dup (p.Ser73LysfsTer2) | rs33969853 | FAM26 | Mother |
|  | c.126_129del (p.Phe42LeufsTer19) | rs80356821 | FAM33 | Father |
|  | c.79G>A (p.Glu27Lys) | rs33950507 | FAM33 | Mother |
|  | c.79G>A (p.Glu27Lys) | rs33950507 | FAM38 | Father |
|  | c.52A>T (p.Lys18Ter) | rs33986703 | FAM38 | Mother |
| *SLC26A4*(1Couple) | c.2086C>T (p.Gln696Ter) | rs752807925 | FAM41 | Father |
|  | c.2086C>T (p.Gln696Ter) | rs752807925 | FAM41 | Mother |
| *USH2A*(1Couple) | c.187C>T (p.Arg63Ter) | rs781223647 | FAM42 | Father |
|  | c.2802T>G (p.Cys934Trp) | rs201527662 | FAM42 | Mother |
